# Pubertal development in girls by breast cancer family history: the LEGACY girls cohort

**DOI:** 10.1186/s13058-017-0849-y

**Published:** 2017-06-08

**Authors:** Mary Beth Terry, Theresa H. M. Keegan, Lauren C. Houghton, Mandy Goldberg, Irene L. Andrulis, Mary B. Daly, Saundra S. Buys, Ying Wei, Alice S. Whittemore, Angeline Protacio, Angela R. Bradbury, Wendy K. Chung, Julia A. Knight, Esther M. John

**Affiliations:** 1 Department of Epidemiology, Columbia University Mailman School of Public Health, 722 West 168th Street, New York, NY 10032 USA; 2 Herbert Irving Comprehensive Cancer Center, Columbia University Medical Center, New York, NY USA; 3 Division of Hematology and Oncology, University of California (UC) Davis School of Medicine, and UC Davis Comprehensive Cancer Center, Sacramento, CA USA; 4 Department of Molecular Genetics, University of Toronto, Toronto, Ontario Canada; 5 Lunenfeld-Tanenbaum Research Institute, Sinai Health System, Toronto, Ontario Canada; 6 Department of Clinical Genetics, Fox Chase Cancer Center, Philadelphia, PA USA; 7 Department of Medicine, University of Utah Health Sciences Center, Huntsman Cancer Institute, Salt Lake City, UT USA; 8 Department of Biostatistics, Columbia University Mailman School of Public Health, New York, NY USA; 9 Departments of Biomedical Data Sciences and Health Research and Policy, and Stanford Cancer Institute, Stanford University School of Medicine, Stanford, CA USA; 10 Departments of Medicine, Hematology/Oncology, Perelman School of Medicine, University of Pennsylvania, Philadelphia, PA USA; 11 Medical Ethics and Health Policy, Perelman School of Medicine, University of Pennsylvania, Philadelphia, PA USA; 12 Departments of Pediatrics and Medicine, Columbia University Medical Center, New York, NY USA; 13 Division of Epidemiology, Dalla Lana School of Public Health, University of Toronto, Toronto, Ontario Canada; 14 Cancer Prevention Institute of California, Fremont, CA USA; 15 Department of Health Research and Policy (Epidemiology), and Stanford Cancer Institute, Stanford University School of Medicine, Stanford, CA USA

**Keywords:** Pubertal development, Menarche, BMI, Breast cancer, Breast cancer family history

## Abstract

**Background:**

Pubertal milestones, such as onset of breast development and menstruation, play an important role in breast cancer etiology. It is unclear if these milestones are different in girls with a first- or second-degree breast cancer family history (BCFH).

**Methods:**

In the LEGACY Girls Study (*n* = 1040), we examined whether three mother/guardian-reported pubertal milestones (having reached Tanner Stage 2 or higher (T2+) for breast and pubic hair development, and having started menstruation) differed by BCFH. We also examined whether associations between body size and race/ethnicity and pubertal milestones were modified by BCFH. We used mother/guardian reports as the primary measure of pubertal milestones, but also conducted sensitivity analyses using clinical Tanner measurements available for a subcohort (*n* = 204). We analyzed cross-sectional baseline data with logistic regression models for the entire cohort, and longitudinal data with Weibull survival models for the subcohort of girls that were aged 5–7 years at baseline (*n* = 258).

**Results:**

BCFH was modestly, but not statistically significantly, associated with Breast T2+ (odds ratio (OR) = 1.36, 95% confidence interval (CI) = 0.88–2.10), with a stronger association seen in the subcohort of girls with clinical breast Tanner staging (OR = 2.20, 95% CI = 0.91–5.32). In a longitudinal analysis of girls who were aged 5–7 years at baseline, BCFH was associated with a 50% increased rate of having early breast development (hazard ratio (HR) = 1.49, 95% CI = 1.0–2.21). This association increased to twofold in girls who were not overweight at baseline (HR = 2.04, 95% CI = 1.29–3.21). BCFH was not associated with pubic hair development and post-menarche status. The median interval between onset of breast development and menarche was longer for BCFH+ than BCFH– girls (2.3 versus 1.7 years), suggesting a slower developmental tempo for BCFH+ girls. Associations between pubertal milestones and body size and race/ethnicity were similar in girls with or without a BCFH. For example, weight was positively associated with Breast T2+ in both girls with (OR = 1.06 per 1 kg, 95% CI = 1.03–1.10) and without (OR = 1.14 per 1 kg, 95% CI = 1.04–1.24) a BCFH.

**Conclusions:**

These results suggest that BCFH may be related to earlier breast development and slower pubertal tempo independent of body size and race/ethnicity.

**Electronic supplementary material:**

The online version of this article (doi:10.1186/s13058-017-0849-y) contains supplementary material, which is available to authorized users.

## Background

In the US, breast cancer incidence, particularly late-stage disease, has been increasing in women under 40 years of age [1–4]. Breast cancer risk factors also have changed over time, with some changes occurring over several generations (e.g., increasing height [5]), and other changes occurring more recently (e.g., declining age at onset of puberty [6]). These trends in risk factors are observed both in the US and globally [7, 8]. While declines in age at menarche began in the early 1900s and stabilized more recently [9, 10], landmark US studies have reported declines in the age at onset of breast development over the last generation [11, 12]. Taller height and heavier weight are two established predictors of earlier pubertal timing [11, 13, 14], but they only partially explain the temporal trends in pubertal timing [15]. Earlier onset of breast development has recently been associated with increased breast cancer risk. In a prospective cohort of 104,931 women, height, age at menarche, and longer interval between onset of breast development and menarche (i.e., slower tempo) were each independently associated with a 20–30% increase in breast cancer risk [16]. As the age at onset of breast development has been declining more rapidly than the age at first menses, the window between these two events has become wider in most populations [17, 18]. Thus, breast cancer incidence may continue to increase, particularly in young women.

Despite these intriguing lines of evidence, limited data exist on whether the timing of pubertal milestones (i.e., age at onset of breast and pubic hair development and first menses) differs between girls with or without a breast cancer family history (BCFH). One exception is the finding that, in girls with a maternal history of breast cancer, risk of early menarche was increased relative to girls without a BCFH (hazard ratio (HR) = 2.2, 95% confidence interval (CI) = 1.0–5.0) [19]. Studies to date have not examined variation in other pubertal development measures by BCFH. If the timing of the individual milestones or tempo between the pubertal events differs by BCFH, then this suggests that the window of susceptibility for environmental exposures and targeted prevention efforts may need to be tailored according to BCFH. If the timing of pubertal milestones is similar between girls with and without a BCFH, then this suggests that pathways related to growth and development and pubertal timing may be equally important in girls with a positive family history (BCFH+) and those without a family history (BCFH–) and therefore potentially modifiable in both groups. We examined whether BCFH was independently related to pubertal milestones after adjusting for race/ethnicity and body mass index (BMI), and whether BCFH modified the associations of body size and race/ethnicity with pubertal milestones.

## Methods

### Study population

The LEGACY (Lessons in Epidemiology and Genetics of Adult Cancer from Youth) Girls Study, a prospective cohort, enrolled 1040 girls at five study sites in the US (New York City, NY; Philadelphia, PA; Salt Lake City, UT; San Francisco Bay Area, CA) and Canada (Toronto, Ontario) from 2011–2013 [20, 21]. These sites comprise the five North American sites of the Breast Cancer Family Registry (BCFR), a multigenerational cohort of breast cancer families (for details, see [22–24]). The girls were primarily between the ages of 6 and 13 years at recruitment, and half had a BCFH. Girls were classified as BCFH+ if the participating mother/guardian reported a BCFH in the daughter’s first- or second-degree relatives or a relative with an identified *BRCA1* or *BRCA2* mutation. Girls were the daughters of BCFR participants or breast cancer patients recruited through regional cancer registries or family genetics and oncology clinics. BCFH– girls were recruited through friend referrals, social media, and community outreach.

### Data collection

We assessed pubertal development and exposures through questionnaires and measurements, with most items collected at baseline and every 6 months thereafter. We administered the Growth and Development Questionnaire to mothers/guardians who assessed their daughter’s sexual maturation using drawings that show five Tanner stages of breast and pubic hair development [25], and reported whether or not their daughter had had her first menses. We also performed clinical breast Tanner staging at the LEGACY sites in New York and Utah. Trained female research staff or a physician completed the visual assessment of breast development according to a standard protocol and assigned a breast Tanner score from 1 to 5. A second score based on both visualization and palpation was recorded for girls when it was difficult to distinguish between breast bud development and fat tissue. In a subset of girls with multiple measures, we previously observed almost perfect agreement between clinical raters for the onset of breast development (Kappas ≥0.94 for Breast T2+ vs. T1) [26]. We used the clinical breast Tanner stage data for sensitivity analyses. Anthropometric measurements, including height and weight, were taken every 6 months by trained research staff. We measured height without shoes using a stadiometer and weight in light clothing using a digital scale. We repeated the height and weight measurements and averaged the two measures.

### Statistical analysis

We assessed the associations of BCFH with the three pubertal outcomes performing logistic regression to determine the odds of being breast and pubic hair Tanner stage 2 or higher (Breast T2+ and Pubic T2+, respectively) vs. 1 (no development yet), and the odds of having experienced menstruation vs. being pre-menarcheal at study enrollment, adjusting for age, BMI, race/ethnicity, and study site. Because of the study design, with multiple siblings enrolled in some families, we used generalized estimating equations (GEE) to account for the correlation between siblings. All analyses using Breast T2+ or Pubic T2+ as an outcome were restricted to girls younger than 13 years because all older girls had mother/guardian-reported Breast T2+ or Pubic T2+. Analyses using first menses as an outcome were restricted to girls 10 years and older because none of the younger girls had started menses as reported by their mother/guardian.

We also estimated median ages at Breast T2+ for each BCFH category using Weibull survival models that were adjusted for the same covariates as in the logistic models. We used bootstrapping with 200 replications to calculate the 95% CI around the medians. In addition, we ran Weibull survival models using prospective data from the subcohort of girls that were 5–7 years old at study enrollment to estimate the associations between BCFH and body size measures with breast development longitudinally. Due to small cell counts, we did not adjust for study site or include girls of mixed race/ethnicity in the longitudinal models.

After examining the association between BCFH and pubertal milestones, we then examined whether the associations of weight, height, and BMI with pubertal milestones were similar in girls with or without a BCFH. To ascertain the influence of BMI on pubertal outcomes, we calculated BMI percentiles by age based on Centers for Disease Control (CDC) growth charts [27]. Girls in the 85th percentile or above for their age were categorized as overweight. Girls with missing data on mother/guardian-reported breast Tanner stage (*n* = 104), pubic hair Tanner stage (*n* = 70), or menarche status (*n* = 24), and girls without body measurements (*n* = 20) were excluded from relevant analyses. We tested for interactions between the main exposures and covariates using a cross-product term in the regression models and the Wald test to assess statistical significance. For the weight and height models, we adjusted for age, race/ethnicity, and study site, and found no statistically significant interactions for any of these variables. We also formally tested for interactions with weight, height, and overweight status and BCFH, and stratified the adjusted models by BCFH. For analyses using the full cohort with BCFH as the exposure, we adjusted for age, BMI at study enrollment, race/ethnicity, and study site and found no interactions for Breast T2+, but a significant interaction between age and BCFH when Pubic T2+ was the outcome. For first menses, the interaction between age and BMI was statistically significant. Interactions by study site for each exposure were not statistically significant. In the longitudinal model for breast development, the interaction between overweight and BCFH was statistically significant. Therefore, we examined the association between BCFH and breast development overall and only for girls that were not overweight.

We conducted supplemental analyses to examine how robust the findings were under different assumptions. First, instead of a categorical BCFH variable, we used the Breast and Ovarian Analysis of Disease Incidence and Carrier Estimation Algorithm (BOADICEA) risk model to estimate lifetime breast cancer risk for each girl [28], by estimating the absolute risk based on family pedigree information. The model produces a continuous score ranging from zero to 100 (data not shown). The scores were used to rank the girls according to their risk of breast cancer and to provide greater precision for the subcohort of BCFH+ girls, but the scores cannot be interpreted as absolute risks because BOADICEA has been validated only for use in adult women [29]. Second, we conducted sensitivity analyses using only data from the two LEGACY sites with information on clinical breast Tanner stage. Based on the validity data from the clinical Tanner scoring (i.e., sensitivity and specificity), we then adjusted the final results for the entire cohort, estimating the adjusted cell counts separately for those with and without a BCFH given the different sensitivity and specificities from our validity studies [26]. Results adjusted for differential reporting are reported in the text, but not in the tables. All analyses were conducted using SAS 9.3, except for Weibull survival analyses that were conducted using StataSE 13 and R 3.3.2.

## Results

**Fig. 1:**
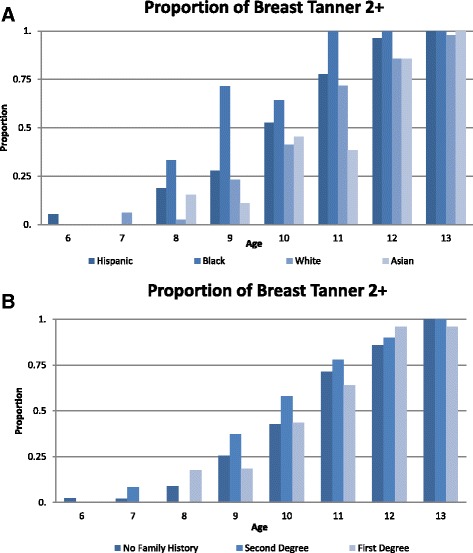
Proportion of breast development by age and race/ethnicity (**a**), and breast cancer family history (**b**)

The proportion of girls who, at study enrollment (baseline), had started breast development (Breast T2+) or pubic hair development (Pubic T2+) or had experienced first menses varied by age, BMI, race/ethnicity, and study site (Table 1). Overall, 45% of girls were Breast T2+, 41% were Pubic T2+, and 19% were post-menarcheal. Girls who had reached pubertal milestones were older, taller, and heavier compared to girls who had not started pubertal development. Figure 1 summarizes the proportion of Breast T2+ by age group and separately by race/ethnicity and BCFH. Black girls were more likely to be Breast T2+ at all ages, but the difference was more pronounced in girls under age 10 years.

**Table 1:** Characteristics of participating girls, by pubertal outcomes; the LEGACY Girls Study

Category	Entire cohort (*n* = 1040)	Breast Tanner, girls aged <13 (*n* = 801)		Pubic hair Tanner, girls aged <13 (*n* = 835)		Menarche, girls ≥10 (*n* = 506)	
		Stage T1 (*n* = 506)	Stage T2+ (*n* = 295)	Stage T1 (*n* = 556)	Stage T2+ (*n* = 279)	No menses (*n* = 313)	Menses (*n* = 193)
Continuous variables, mean (SD)							
Age (years)	10.1 (2.4)	8.7 (1.7)	11.2 (1.3)	8.7 (1.7)	11.2 (1.4)	11.4 (1.0)	13.2 (1.2)
Weight (kg)	37.8 (14.6)	29.6 (8.4)	46.0 (12.4)	30.1 (9.4)	44.7 (13.2)	41.1 (10.8)	55.5 (13.1)
Height (cm)	142.6 (15.3)	133.5 (11.0)	151.8 (10.7)	133.4 (11.1)	151.2 (11.1)	149.9 (9.2)	161.2 (7.5)
BMI (kg/m^2^)	18.0 (4.3)	16.4 (3.4)	19.8 (4.3)	16.7 (3.7)	19.3 (4.3)	18.2 (3.8)	21.3 (4.3)
Maternal age at menarche	12.7 (1.5)	12.9 (1.5)	12.4 (1.5)	12.8 (1.5)	12.6 (1.5)	12.9 (1.5)	12.4 (1.5)
Maternal age at daughter’s birth	32.3 (5.5)	32.6 (5.4)	31.8 (5.7)	32.5 (5.4)	32.0 (5.7)	32.4 (5.2)	31.2 (5.8)
Categorical variables, count (%)							
Overweight							
≥ 85th percentile	193 (18.6)	60 (11.9)	90 (30.5)	81 (14.6)	71 (25.5)	46 (14.7)	55 (28.5)
Race/ethnicity							
Hispanic	193 (18.6)	79 (15.6)	60 (20.3)	88 (15.8)	60 (21.5)	44 (14.1)	58 (30.1)
Black	78 (7.5)	27 (5.3)	34 (11.5)	27 (4.9)	36 (12.9)	22 (7.0)	17 (8.8)
Non-hispanic white	646 (62.1)	343 (67.8)	172 (58.3)	371 (66.7)	160 (57.4)	209 (66.8)	91 (47.2)
Asian	93 (8.9)	46 (9.1)	19 (6.4)	55 (9.9)	16 (5.7)	31 (9.9)	19 (9.4)
Mixed race/ethnicity	30 (2.9)	11 (2.2)	10 (3.4)	15 (2.7)	7 (2.5)	7 (2.2)	8 (4.2)
Study site							
Philadelphia	154 (14.8)	72 (14.2)	53 (18.0)	74 (13.3)	52 (18.6)	63 (20.1)	25 (13.0)
Utah	164 (15.8)	86 (17.0)	32 (10.9)	100 (18.0)	30 (10.8)	45 (14.4)	16 (8.3)
New York	168 (16.2)	95 (18.8)	45 (15.2)	102 (18.4)	38 (13.6)	39 (12.5)	28 (14.5)
Ontario, Canada	192 (18.5)	103 (20.4)	53 (18.0)	113 (20.3)	49 (17.6)	53 (16.9)	37 (19.2)
San Francisco Bay Area	362 (34.8)	150 (29.6)	112 (38.0)	167 (30.0)	110 (39.4)	113 (36.1)	87 (45.1)
Maternal education							
Some college/vocational/technical school or less	284 (27.3)	114 (22.5)	101 (35.1)	139 (25.0)	85 (30.5)	77 (24.6)	73 (37.8)
Bachelor’s degree	377 (36.3)	194 (38.3)	85 (29.5)	210 (37.8)	90 (32.3)	107 (34.2)	62 (32.1)
Graduate degree	353 (33.9)	191 (37.8)	102 (35.4)	199 (35.8)	98 (35.1)	124 (39.6)	45 (23.3)
Breast cancer family history (BCFH)							
No BCFH	497 (47.8)	267 (52.7)	142 (48.1)	277 (49.8)	146 (52.3)	154 (49.2)	84 (43.5)
Any BCFH	533 (51.3)	239 (47.2)	153 (51.9)	279 (50.2)	133 (47.7)	159 (50.8)	109 (56.5)
First-degree BCFH	219 (21.1)	89 (17.6)	64 (21.7)	99 (17.8)	63 (22.6)	71 (22.7)	53 (27.5)
Second-degree BCFH Only	313 (30.1)	150 (29.6)	89 (30.2)	180 (32.4)	70 (25.1)	88 (28.1)	56 (29.0)

*BMI* body mass index, *SD* standard deviation.

BCFH+ girls were more likely to be Breast T2+ than BCFH– girls, but this association was not statistically significant (odds ratio (OR) = 1.36, 95% CI = 0.88–2.10 adjusted for age, BMI, race/ethnicity, and study site) (Table 2). The positive association between Breast T2+ and BCFH was limited to girls with a second-degree BCFH (adjusted OR = 1.8, 95% CI = 1.07–3.05). When we further examined Model 2 presented in Table 2 using a BOADICEA risk score, the results were also consistent with earlier breast development in girls with a higher score (data not shown). In the subcohort of LEGACY girls with clinical breast Tanner staging (*n* = 204), the ORs (95% CI) for Breast T2+ after adjusting for age, BMI, and study site were 2.20 (0.91–5.32), 1.78 (0.41–7.69), and 2.27 (0.82–6.25) for BCFH+, first-degree BCFH+, and second-degree BCFH+ girls, respectively (data not shown). We did not observe an association between BCFH and pubic hair development or age at menarche (adjusted OR = 0.81, 95% CI = 0.55–1.20 and adjusted OR = 0.69, 95% CI = 0.38–1.23, respectively) (Table 2).

**Table 2:** Associations of measures of breast cancer family history (BCFH) with pubertal outcomes; LEGACY Girls Study

	Odds ratio (95% confidence interval)					
	Breast Tanner stage T2 + ^a^		Pubic hair Tanner stage T2 + ^a^		Post-menarche status^b^	
Measures of BCFH^c^	Model 1^d^	Model 2^e^	Model 1^d^	Model 2^e^	Model 1^d^	Model 2^f^
	*n* = 801	*n* = 786	*n* = 835	*n* = 819	*n* = 506	*n* = 493
Any BCFH	1.35 (0.92–1.98)	1.36 (0.88–2.10)	0.83 (0.58–1.20)	0.81 (0.55–1.20)	0.69 (0.42–1.14)	0.69 (0.38–1.23)
First-degree BCFH	1.05 (0.63–1.75)	0.99 (0.58–1.68)	0.98 (0.60–1.58)	0.94 (0.58–1.55)	0.66 (0.35–1.25)	0.67 (0.32–1.38)
Second-degree BCFH Only	1.60 (1.03–2.50)	1.80 (1.07–3.05)	0.76 (0.50–1.16)	0.75 (0.48–1.17)	0.74 (0.41–1.32)	0.72 (0.35–1.47)

^a^Models exclude girls aged ≥13 years (*n* = 135)

^b^Excludes girls aged <10 years (*n* = 510)

^c^No BCFH is the referent group for all models

^d^Adjusted for age

^e^Adjusted for age, body mass index (BMI), race/ethnicity, and study site

^f^Adjusted for age, BMI, race/ethnicity, site, and BMI × age

Further analyses in the subcohort of girls with clinical Tanner staging showed that the difference in breast Tanner stage by degree of BCFH was driven by measurement error in maternal Tanner reporting that differed by degree of BCFH [26]. Compared to the clinical breast Tanner staging, the sensitivity of maternal breast Tanner report was 68.4% for first-degree BCFH, 77.8% for second-degree BCFH, and 69.4% for no BCFH. This means that mothers with breast cancer were less likely to report that their daughters have started breast development compared to mothers with a BCFH, but who themselves did not have breast cancer. The specificities were 93.8%, 97.8%, and 92.8%, respectively. In the overall LEGACY cohort, adjustment for sensitivity and specificity of maternal breast Tanner reporting by degree of BCFH altered the overall association of Breast T2+ with BCFH very little (OR = 1.2, 95% CI = 0.9–1.7), but strengthened the association with first-degree BCFH (OR = 1.6, 95% CI = 1.1–2.4). No association remained with second-degree BCFH after adjustment for sensitivity and specificity (OR = 1.1, 95% CI = 0.8–1.5). Consistent with these results from the subcohort of girls with clinical Tanner staging, the overall cohort analyses suggest that girls with a BCFH may have earlier breast development. When we considered the interval between onset of breast development and first menses (i.e., tempo), we found that the median interval was longer for girls with a BCFH (2.3 years, 95% CI = 1.5–3.1) compared to girls without a BCFH (1.7 years, 95% CI = 1.3–2.1) (Additional file 1: Table S1). The difference in tempo between girls with or without a BCFH was 0.6 years (95% CI = −0.30 to 1.51), but was not statistically significant.

**Fig. 2:**
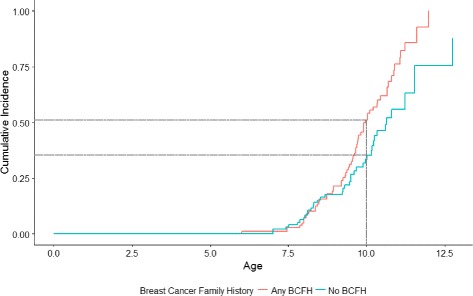
Kaplan-Meier cumulative incidence of breast development by breast cancer family history (*BCFH*) among girls aged 5–7 years at first visit with a BMI <85th percentile

In a longitudinal analysis of girls who were 5–7 years at baseline, BCFH was associated with a 50% higher rate of early breast development (HR = 1.49, 95% CI = 1.0–2.21 adjusted for age, BMI, and race/ethnicity). This association increased to twofold in girls who were not overweight at baseline (adjusted HR = 2.04, 95% CI = 1.29–3.21) (Table 3). In girls who were not overweight (BMI <85th percentile), 51.2% of BCFH+ girls experienced breast development by age 10 years, compared to 33.5% for BCFH– (Fig. 2).

**Table 3:** Associations of measures of breast cancer family history (BCFH) with breast development among girls aged 5–7 years at first visit using Weibull longitudinal models; LEGACY Girls Study

	Hazard ratio (95% confidence interval)			
	All girls aged 5–7 years at first visit		Girls aged 5–7 years at first visit, BMI <85th percentile	
Measures of BCFH^a^	Model 1^b^	Model 2^c, d^	Model 1^b^	Model 2^c, d^
	*n* = 258	*n* = 248	*n* = 210	*n* = 206
Any BCFH	1.33 (0.93–1.91)	1.49 (1.00–2.21)	1.69 (1.12–2.55)	2.04 (1.29–3.21)
First-degree BCFH	1.28 (0.83–1.98)	1.24 (0.76–2.03)	1.88 (1.13–3.12)	2.21 (1.31–3.73)
Second-degree BCFH only	1.38 (0.93–2.04)	1.68 (1.09–2.59)	1.63 (1.03–2.56)	1.94 (1.17–3.24)

^a^No BCFH is the referent group for all models

^b^Adjusted for age as the underlying time scale

^c^Adjusted for age as the underlying time scale, body mass index at baseline, and race/ethnicity

^d^Girls with mixed race/ethnicity were excluded due to small cell counts

Weight and overweight (BMI ≥85th percentile for age) were positively associated with being Breast T2+ and post-menarcheal, adjusting for age, height, race/ethnicity, and study site (Table 4). For example, for each kilogram increase in weight, there was a 9% increase in the odds of girls having started breast development (adjusted OR = 1.09, 95% CI = 1.05–1.12). Height was associated with pubic hair development (adjusted OR = 1.06, 95% CI = 1.03–1.10 per centimeter). Even after controlling for age, weight, and height, black race was associated with being Breast T2+ (OR = 3.88, 95% CI = 1.74–8.65) and Pubic T2+ (OR = 5.27, 95% CI = 2.38–11.65); Asian race was associated with a lower odds of being Pubic T2+ (OR = 0.44, 95% CI = 0.21–0.95); and Hispanic ethnicity was associated with being post-menarcheal (OR = 2.41, 95% CI = 1.15–5.04) compared with non-Hispanic whites.

**Table 4:** The associations of weight and height with pubertal outcomes, by breast cancer family history (BCFH)

	Odds ratio (95% confidence interval)		
	Overall	BCFH+ girls	BCFH– girls
Breast Tanner stage T2 + ^a^			
* n*	786	383	403
Model 1^c^			
Weight (per 1 kg)	1.09 (1.05–1.12)	1.06 (1.03–1.10)	1.14 (1.04–1.24)
Height (per 1 cm)	1.03 (0.99–1.07)	1.03 (0.99–1.08)	1.01 (0.94–1.09)
Race/ethnicity			
Non-Hispanic white	1.00 (reference)	1.00 (reference)	1.00 (reference)
Asian	0.55 (0.22–1.41)	0.66 (0.18–2.37)	0.55 (0.16–1.86)
Black	3.88 (1.74–8.65)	5.04 (1.21–20.94)	3.80 (1.43–10.11)
Hispanic	1.18 (0.65–2.13)	1.34 (0.58–3.14)	0.91 (0.37–2.25)
Mixed race/ethnicity	2.54 (0.75–8.54)	7.61 (0.11–533.98)	1.81 (0.56–5.78)
Model 2^d^			
Overweight (≥85th percentile)	4.65 (2.58–8.38)	3.32 (1.43–7.69)	6.72 (2.70–16.74)
Pubic hair Tanner stage T2 + ^a^			
* n*	819	403	416
Model 1^c^			
Weight (per 1 kg)	1.02 (0.99–1.05)	1.02 (0.98–1.06)	1.02 (0.98–1.06)
Height (per 1 cm)	1.06 (1.03–1.10)	1.05 (1.00–1.11)	1.07 (1.02–1.12)
Race/ethnicity			
Non-Hispanic white	1.00 (reference)	1.00 (reference)	1.00 (reference)
Asian	0.44 (0.21–0.95)	0.39 (0.13–1.20)	0.46 (0.17–1.24)
Black	5.27 (2.38–11.65)	9.58 (2.53–36.19)	3.88 (1.48–10.17)
Hispanic	1.55 (0.85–2.84)	1.71 (0.65–4.47)	1.36 (0.60–3.11)
Mixed race/ethnicity	1.46 (0.37–5.77)	2.45 (0.54–11.05)	1.09 (0.16–7.53)
Model 2^d^			
Overweight (≥85th percentile)	1.24 (0.75–2.06)	1.26 (0.56–2.83)	1.40 (0.71–2.75)
Post-menarche status^b^			
* n*	493	260	233
Model 1^c^			
Weight (per 1 kg)	1.08 (1.04–1.12)	1.09 (1.03–1.16)	1.06 (1.00–1.12)
Height (per 1 cm)	1.03 (0.99–1.08)	0.98 (0.93–1.05)	1.09 (1.03–1.16)
Race/ethnicity			
Non-Hispanic white	1.00 (reference)	1.00 (reference)	1.00 (reference)
Asian	1.06 (0.46–2.45)	0.52 (0.15–1.81)	1.60 (0.46–5.58)
Black	1.69 (0.65–4.40)	1.37 (0.34–5.51)	2.07 (0.50–8.54)
Hispanic	2.41 (1.15–5.04)	1.22 (0.49–3.09)	8.08 (2.24–29.19)
Mixed race/ethnicity	2.21 (0.20–23.96)	0.29 (0.02–3.51)	5.28 (0.06–480.69)
Model 2^d^			
Overweight (≥85th percentile)	4.19 (2.15–8.14)	4.75 (2.08–10.82)	3.73 (1.21–11.51)

^a^Excludes girls aged ≥13 years (*n* = 135)

^b^Excludes girls aged <10 years (*n* = 510)

^c^Model includes weight, height, race/ethnicity, and is adjusted for age and study site. Non-Hispanic white is the referent race/ethnicity

^d^Model includes overweight and is adjusted for height, race/ethnicity, age, and study site. Non-Hispanic white is the referent race/ethnicity

When we stratified the logistic regression models by BCFH, the odds of reaching each pubertal milestone were influenced by BMI and race/ethnicity in both BCFH+ and BCFH– girls (Table 4), but the associations were stronger in BCFH– girls. In both groups of girls, BMI (as reflected by *z* scores) increased with higher breast Tanner stage and was higher for girls at Breast T2+ than for those at Breast T1, but the means were generally larger for the BCFH– girls (Additional file 2: Figure S1). The associations between height and Pubic T2+ and between weight and post-menarche status were similar for BCFH+ and BCFH– girls (Table 4). Height was associated with being post-menarcheal in BCFH– girls only (adjusted OR = 1.09, 95% CI = 1.03–1.16 per centimeter). We did not find statistically significant interactions between BCFH and weight, overweight, or height for any of the pubertal outcomes (*p* > 0.05), with the exception of BCFH and height in relation to being post-menarcheal (*p* = 0.02). Longitudinal analyses also supported associations between body size and timing of pubertal milestones in both girls with and without a BCFH (Table 5).The interaction between overweight and BCFH was significant in the longitudinal analysis (*p* < 0.05), with a stronger association for BCFH– girls.

**Table 5:** The associations of weight and height with breast development among girls aged 5–7 years old at first visit using Weibull longitudinal models; LEGACY Girls Study

	Hazard ratio (95% confidence interval)		
	Overall	BCFH+ girls	BCFH– girls
Breast Tanner stage T2+			
* n*	248	127	121
Model 1^a^			
Weight (per 1 kg)	1.05 (1.03–1.08)	1.05 (1.02–1.08)	1.10 (1.03–1.17)
Height (per 1 cm)	1.01 (0.98–1.04)	0.99 (0.95–1.02)	0.98 (0.93–1.04)
Race/ethnicity			
Non-Hispanic white	1 (reference)	1 (reference)	1 (reference)
Asian	1.12 (0.54–2.35)	1.18 (0.35–3.98)	1.31 (0.53–3.25)
Black	1.31 (0.73–2.37)	1.36 (0.76–2.42)	1.51 (0.56–4.08)
Hispanic	1.52 (0.95–2.44)	2.75 (1.48–5.11)	0.88 (0.37–2.08)
Model 2^b^			
Overweight (≥85th percentile)	3.61 (2.16–6.02)	2.02 (0.99–4.11)	9.40 (4.94–17.91)

*BCFH* breast cancer family history

^a^Model is adjusted for age as the underlying time scale and includes weight, height, and race/ethnicity. Mixed race/ethnicity is excluded due to small cell counts

^b^Model is adjusted for age as the underlying time scale and includes overweight, height, and race/ethnicity. Mixed race/ethnicity is excluded due to small cell counts

## Discussion

We observed that BCFH, independent of body size and race/ethnicity, was related to earlier breast development, but not to early pubarche or menarche. The findings were modest, and not statistically significant in the cross-sectional analyses of the overall cohort analyzing baseline data, but were stronger and statistically significant in the longitudinal analyses of the subcohort of girls who were 5–7 years old at baseline. We also observed stronger associations in the subcohort of girls where breast development was measured through clinical Tanner rather than maternal/guardian report. In cross-sectional analyses, we also found that higher weight and overweight (BMI ≥85th percentile for age) were associated with having started breast development and menstruation, whereas taller height was associated with having started pubic hair development. The timing of pubertal milestones also varied by race/ethnicity. The associations between weight, overweight, and height and pubertal milestones were seen in girls with and without a BCFH.

While menarche status and pubic hair development were similar in girls with or without a BCFH, our findings suggest that onset of puberty, marked by the beginnings of breast development, may be earlier in BCFH+ girls. We observed modest differences in timing of breast development by degree of BCFH. The differences between girls with a first- or second-degree BCFH were likely driven by maternal differences in breast Tanner reporting, as the associations by degree of BCFH were stronger for clinical Tanner assessment than maternal assessment and were similar to each other in magnitude when we examined the subcohort of girls with clinical breast Tanner staging. The clinical breast Tanner staging results are unlikely to be driven by measurement error given the high reliability between clinical raters in the study [26]. The consistency of the clinical Tanner results with those from the maternal assessment after adjustment for measurement error suggests a modest association between BCFH and earlier breast development. Modest associations between BCFH and the timing of any individual pubertal milestones may indicate a slower pubertal tempo, especially since the results suggest that a BCFH may accelerate breast development. Even though the age at menarche did not differ by BCFH, the earlier breast development in girls with a BCFH translated into differences in pubertal tempo measured in cross-sectional analyses which was 2.3 years in girls with any BCFH and 1.7 years in BCFH– girls. Longer follow-up will be needed to confirm if these differences are statistically significant in longitudinal analyses as the subcohort of girls we followed prospectively for onset of breast development was too young to have precision for estimates of age at menarche. The Breakthrough Generations Study found that a pubertal tempo of more than 2 years was associated with a 27% higher risk of breast cancer [16]. Thus, the BCFH+ girls with an average tempo greater than 2 years and at least 0.5 years longer than that of BCFH– girls may be at higher breast cancer risk from this wider window as well as other factors. Furthermore, longitudinal analyses of girls who were 5–7 years at baseline support an earlier onset of breast development in BCFH+ girls compared with BCFH– girls.

There is emerging and compelling evidence from large genome-wide association studies (GWAS) that specific genetic variants are associated with growth in height during puberty, BMI, and age at menarche; many of the variants that are related to height and pubertal outcomes have also been associated with breast cancer risk. For example, GWAS have found at least 180 single nucleotide polymorphisms (SNPs) that are associated with height; of these, 168 are also associated with breast cancer risk, as well as age at menarche, weight, and BMI [30–33]. Five genes (*MAPK3*, *PXMP3*, *VGLL3*, *ADCY3/POMC*, and *LIN28B*) have been associated with pubertal timing, affecting tempo and timing of growth before and during puberty [34, 35]. *ADCY3/POMC* has been implicated in obesity among children and adults [36, 37]. Interestingly, many of these same genes, including *MAPK3*, are also associated with breast cancer risk [38–40]. Variants in the *IGF* signaling pathway have been associated with height [41–44] and, recently, *LIN28B* has been shown to regulate the miRNA let-7 family, which in turn affects the IGF signaling pathway in the head and neck and other cancers [39, 45]. If BCFH+ girls are more likely to have a greater clustering of genetic variants in these genes compared to BCFH– girls, then these genes may account for some of the associations we observed between BCFH and onset of breast development.

Our study has several strengths, including the racial/ethnic diversity of the study population, the measurement of weight and height rather than relying on self-report, and the collection of several indices of pubertal development. Furthermore, we recruited girls across a wide spectrum of breast cancer risk and collected detailed data on first- and second-degree BCFH, both on the maternal and paternal side. This allowed us to examine whether pubertal development varies by extent of BCFH. We acknowledge that BCFH changes over time and we regularly update this information in our cohort. However, we cannot exclude the possibility of misclassification of BCFH. The longitudinal analysis we included, however, defines BCFH for a specific time window based on the daughter’s age at baseline. Our cohort also is the only youth cohort worldwide that is enriched for girls with a BCFH and is the first to assess the relation between early-life exposures and pubertal development in girls who are at increased risk of developing breast cancer due to their family history. Our LEGACY cohort complements other youth cohorts, such as the Breast Cancer Environmental Research Program (BCERP) cohort [46], which includes only a small proportion of girls from families with breast cancer. Women with a BCFH are more likely to be diagnosed with premenopausal breast cancer [2], and early-life exposures are more strongly associated with premenopausal than postmenopausal breast cancer risk [47]. Thus it is essential to have a cohort like ours that can evaluate the role of early-life factors across the continuum of risk to inform breast cancer prevention. Environmental factors are important modifiers of breast cancer risk, even within the 5–30% of cancers attributed to familial clustering [48, 49]. Our work in adults suggests that, even in high-risk families, biomarkers such as DNA repair phenotype and DNA methylation, which are known to differ across the life course based on environmental exposures, differ between affected and unaffected sisters [50–52].

## Conclusions

These results suggest that BCFH may be related to earlier onset of breast development independent of body size and race/ethnicity, and support the premise that body size is an important predictor of pubertal milestones even in girls with a BCFH. The findings from our study also suggest that girls with a BCFH may have a slower tempo between onset of breast development and menarche, indicating a wider window of breast cancer susceptibility.

## Additional files

Additional file 1: Table S1. Associations of measures of breast cancer family history (BCFH) with age at onset and pubertal tempo. (DOCX 14 kb)

Additional file 2: Figure S1. Mean BMI *z* scores and 95% confidence intervals stratified by age group, breast Tanner stage, and breast cancer family history (BCFH). (PDF 217 kb)

## Abbreviations

BCFH: Breast cancer family history
BMI: Body mass index
CI: Confidence interval
HR: Hazard ratio
OR: Odds ratio

## References

[1] Johnson RH, Chien FL, Bleyer A (2013). Incidence of breast cancer with distant involvement among women in the United States, 1976 to 2009. JAMA.

[2] Colditz GA, Rosner BA, Speizer FE (1996). Risk factors for breast cancer according to family history of breast cancer. For the Nurses’ Health Study Research Group. J Natl Cancer Inst.

[3] Sturtz LA, Melley J, Mamula K, Shriver CD, Ellsworth RE (2014). Outcome disparities in African American women with triple negative breast cancer: a comparison of epidemiological and molecular factors between African American and Caucasian women with triple negative breast cancer. BMC Cancer.

[4] Boyle P (2012). Triple-negative breast cancer: epidemiological considerations and recommendations. Ann Oncol.

[5] Onland-Moret NC, Peeters PHM, van Gils CH (2005). Age at menarche in relation to adult height: the EPIC study. Am J Epidemiol.

[6] Biro FM, Greenspan LC, Galvez MP (2012). Puberty in girls of the 21st century. J Pediatr Adolesc Gynecol.

[7] Schooling CM, Hui LL, Ho LM, Lam T-H, Leung GM (2012). Cohort profile: “children of 1997”: a Hong Kong Chinese birth cohort. Int J Epidemiol.

[8] Aksglaede L, Sørensen K, Petersen JH, Skakkebaek NE, Juul A (2009). Recent decline in age at breast development: the Copenhagen Puberty Study. Pediatrics.

[9] Euling SY, Herman-Giddens ME, Lee PA (2008). Examination of US puberty-timing data from 1940 to 1994 for secular trends: panel findings. Pediatrics.

[10] Herman-Giddens ME, Kaplowitz PB, Wasserman R (2004). Navigating the recent articles on girls’ puberty in Pediatrics: what do we know and where do we go from here?. Pediatrics.

[11] Biro FM, Greenspan LC, Galvez MP (2013). Onset of breast development in a longitudinal cohort. Pediatrics.

[12] Herman-Giddens ME, Slora EJ, Wasserman RC (1997). Secondary sexual characteristics and menses in young girls seen in office practice: a study from the Pediatric Research in Office Settings network. Pediatrics.

[13] Yermachenko A, Dvornyk V. Nongenetic determinants of age at menarche: a systematic review. Biomed Res Int. 2014;2014:371583. http://www.pubmedcentral.nih.gov/articlerender.fcgi?artid=4094877&tool=pmcentrez&rendertype=abstract. doi:10.1155/2014/371583. Accessed 3 Mar 2016. 10.1155/2014/371583 PMC4094877 25050345

[14] Davison KK, Susman EJ, Birch LL (2003). Percent body fat at age 5 predicts earlier pubertal development among girls at age 9. Pediatrics.

[15] Kaplowitz PB, Slora EJ, Wasserman RC, Pedlow SE, Herman-Giddens ME (2001). Earlier onset of puberty in girls: relation to increased body mass index and race. Pediatrics.

[16] Bodicoat DH, Schoemaker MJ, Jones ME (2014). Timing of pubertal stages and breast cancer risk: the Breakthrough Generations Study. Breast Cancer Res.

[17] de Muinich Keizer SM, Mul D (2001). Trends in pubertal development in Europe. Hum Reprod Update.

[18] Kaplowitz P (2006). Pubertal development in girls: secular trends. Curr Opin Obstet Gynecol.

[19] Epplein M, Novotny R, Daida Y, Vijayadeva V, Onaka AT, Le Marchand L (2010). Association of maternal and intrauterine characteristics with age at menarche in a multiethnic population in Hawaii. Cancer Causes Control.

[20] John EM, Terry MB, Keegan THM, et al. The LEGACY Girls Study: growth and development in the context of breast cancer family history. Epidemiol. 2016;27(3):438–48. http://www.ncbi.nlm.nih.gov/pubmed/26829160. doi:10.1097/EDE.0000000000000456. Accessed 3 Mar 2016. 10.1097/EDE.0000000000000456 PMC5341739 26829160

[21] The LEGACY Girls Study. www.legacygirlsstudy.org/. Accessed 14 Apr 2016.

[22] Terry MB, Phillips K-A, Daly MB, et al. Cohort Profile: The Breast Cancer Prospective Family Study Cohort (ProF-SC). Int J Epidemiol. 2016;45(3):683–92. doi:10.1093/ije/dyv118. 10.1093/ije/dyv118 PMC5005937 26174520

[23] John EM, Hopper JL, Beck JC (2004). The Breast Cancer Family Registry: an infrastructure for cooperative multinational, interdisciplinary and translational studies of the genetic epidemiology of breast cancer. Breast Cancer Res.

[24] Breast Cancer Family Registry. http://www.bcfamilyregistry.org/. Accessed 14 Apr 2016.

[25] Marshall WA, Tanner JM (1969). Variations in pattern of pubertal changes in girls. Arch Dis Child.

[26] Terry MB, Goldberg M, Schechter S (2016). Comparison of clinical, maternal, and self pubertal assessments: implications for health studies. Pediatr.

[27] Centers for Disease Control and Prevention National Center for Health Statistics. Individual growth charts. http://www.cdc.gov/growthcharts/charts.htm. Accessed 14 Apr 2016.

[28] Antoniou AC, Pharoah PPD, Smith P, Easton DF (2004). The BOADICEA model of genetic susceptibility to breast and ovarian cancer. Br J Cancer.

[29] MacInnis RJ, Bickerstaffe A, Apicella C (2013). Prospective validation of the breast cancer risk prediction model BOADICEA and a batch-mode version BOADICEACentre. Br J Cancer.

[30] Nickels S, Truong T, Hein R (2013). Evidence of gene-environment interactions between common breast cancer susceptibility loci and established environmental risk factors. PLoS Genet.

[31] Schoeps A, Rudolph A, Seibold P (2014). Identification of new genetic susceptibility loci for breast cancer through consideration of gene-environment interactions. Genet Epidemiol.

[32] Barrdahl M, Canzian F, Joshi AD (2014). Post-GWAS gene-environment interplay in breast cancer: results from the Breast and Prostate Cancer Cohort Consortium and a meta-analysis on 79,000 women. Hum Mol Genet.

[33] Zhang B, Shu XO, Delahanty RJ, et al. Height and breast cancer risk: evidence from prospective studies and mendelian randomization. J Natl Cancer Inst. 2015;107(11). doi:10.1093/jnci/djv219. 10.1093/jnci/djv219 PMC4643630 26296642

[34] Cousminer DL, Berry DJ, Timpson NJ (2013). Genome-wide association and longitudinal analyses reveal genetic loci linking pubertal height growth, pubertal timing and childhood adiposity. Hum Mol Genet.

[35] Sovio U, Mook-Kanamori DO, Warrington NM (2011). Association between common variation at the FTO locus and changes in body mass index from infancy to late childhood: the complex nature of genetic association through growth and development. PLoS Genet.

[36] Bradfield JP, Taal HR, Timpson NJ (2012). A genome-wide association meta-analysis identifies new childhood obesity loci. Nat Genet.

[37] Speliotes EK, Willer CJ, Berndt SI (2010). Association analyses of 249,796 individuals reveal 18 new loci associated with body mass index. Nat Genet.

[38] Easton DF, Pooley KA, Dunning AM (2007). Genome-wide association study identifies novel breast cancer susceptibility loci. Nature.

[39] Zhou J, Ng S-B, Chng W-J (2013). LIN28/LIN28B: an emerging oncogenic driver in cancer stem cells. Int J Biochem Cell Biol.

[40] He C, Chasman DI, Dreyfus J (2012). Reproductive aging-associated common genetic variants and the risk of breast cancer. Breast Cancer Res.

[41] Hankinson SE, Willett WC, Colditz GA (1998). Circulating concentrations of insulin-like growth factor-I and risk of breast cancer. Lancet.

[42] Kansra AR, Dolan LM, Martin LJ, Deka R, Chernausek SD (2012). IGF receptor gene variants in normal adolescents: effect on stature. Eur J Endocrinol.

[43] Juul A, Bang P, Hertel NT (1994). Serum insulin-like growth factor-I in 1030 healthy children, adolescents, and adults: relation to age, sex, stage of puberty, testicular size, and body mass index. J Clin Endocrinol Metab.

[44] Schernhammer ES, Holly JM, Pollak MN, Hankinson SE (2005). Circulating levels of insulin-like growth factors, their binding proteins, and breast cancer risk. Cancer Epidemiol Biomarkers Prev.

[45] Alajez NM, Shi W, Wong D, et al. Lin28b promotes head and neck cancer progression via modulation of the insulin-like growth factor survival pathway. Oncotarget. 2012;3(12):1641–52. http://www.pubmedcentral.nih.gov/articlerender.fcgi?artid=3681501&tool=pmcentrez&rendertype=abstract. doi:10.18632/oncotarget.785. Accessed 14 May 2015. 10.18632/oncotarget.785 PMC3681501 23482325

[46] Biro FM, Galvez MP, Greenspan LC (2010). Pubertal assessment method and baseline characteristics in a mixed longitudinal study of girls. Pediatrics.

[47] Colditz GA, Frazier AL (1995). Models of breast cancer show that risk is set by events of early life: prevention efforts must shift focus. Cancer Epidemiol Biomarkers Prev.

[48] Becher H, Schmidt S, Chang-Claude J (2003). Reproductive factors and familial predisposition for breast cancer by age 50 years. A case-control family study for assessing main effects and possible gene-environment interaction. Int J Epidemiol.

[49] Delgado-Cruzata L, Wu H-C, Liao Y, Santella RM, Terry MB (2014). Differences in DNA methylation by extent of breast cancer family history in unaffected women. Epigenetics.

[50] Machella N, Terry MB, Zipprich J (2008). Double-strand breaks repair in lymphoblastoid cell lines from sisters discordant for breast cancer from the New York site of the BCFR. Carcinogenesis.

[51] Zipprich J, Terry MB, Liao Y (2009). Plasma protein carbonyls and breast cancer risk in sisters discordant for breast cancer from the New York site of the Breast Cancer Family Registry. Cancer Res.

[52] Wu H-C, Delgado-Cruzata L, Machella N, Wang Q, Santella RM, Terry MB (2013). DNA double-strand break repair genotype and phenotype and breast cancer risk within sisters from the New York site of the Breast Cancer Family Registry (BCFR). Cancer Causes Control.

