# Supplementary material for: Pubertal development in girls by breast cancer family history: the LEGACY girls cohort
Source: Breast Cancer Res. 2017 Jun 8;19:69. doi: 10.1186/s13058-017-0849-y (PMC5465536; doi:10.1186/s13058-017-0849-y)
Supplement: Supplementary file 1 — Associations of measures of breast cancer family history (BCFH) with age at onset and pubertal tempo. (DOCX 14 kb) [file 13058_2017_849_MOESM1_ESM.docx]

Table S1. Associations of Measures of Breast Cancer Family History (BCFH) with Age at Onset and Pubertal Tempo

|  | Ages (Median, 10^th^, 90^th^ percentiles) at Onset (Years) | | | | Tempo (Years)^A^ | |
| --- | --- | --- | --- | --- | --- | --- |
| Measures of BCFH | Thelarche (T2+)^B,D^ | | Menarche^C,D^ | | Thelarche to Menarche | |
|  | Median | 95% CI | Median | 95% CI | Median | 95% CI |
| No BCFH | 10.9 | (10.6, 11.2) | 12.6 | (12.4, 12.9) | 1.7 | (1.3, 2.1) |
| Any BCFH | 10.6 | (10.1, 11.1) | 12.9 | (12.3, 13.5) | 2.3 | (1.5, 3.1) |
|  |  |  |  |  |  |  |
| First-degree BCFH | 10.9 | (9.0, 12.7) | 13.5 | (11.4, 15.6) | 2.6 | (0.03, 5.2) |
|  |  |  |  |  |  |  |
|  |  |  |  |  |  |  |
| Second-degree BCFH Only | 10.4 | (6.3-14.6) | 12.8 | (11.6, 14.1) | 2.4 | (-1.8, 6.6) |

^A^ Tempo is defined as the difference in the estimated median age at onset of pubertal events

^B^ Models exclude girls ages ≥13 years (n=135)

^C^ Excludes girls ages <10 years (n=510)

^D^ Adjusted for BMI, race/ethnicity, and study site
