# Supplementary figures and images for: Pubertal development in girls by breast cancer family history: the LEGACY girls cohort
Source: Breast Cancer Res. 2017 Jun 8;19:69. doi: 10.1186/s13058-017-0849-y (PMC5465536; doi:10.1186/s13058-017-0849-y)

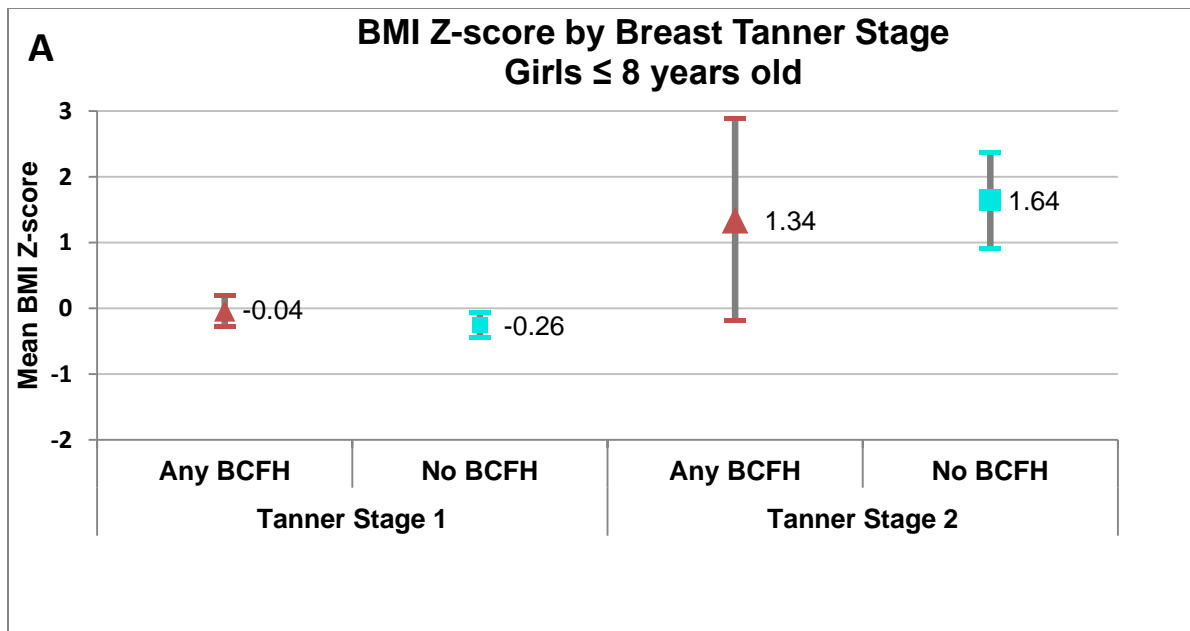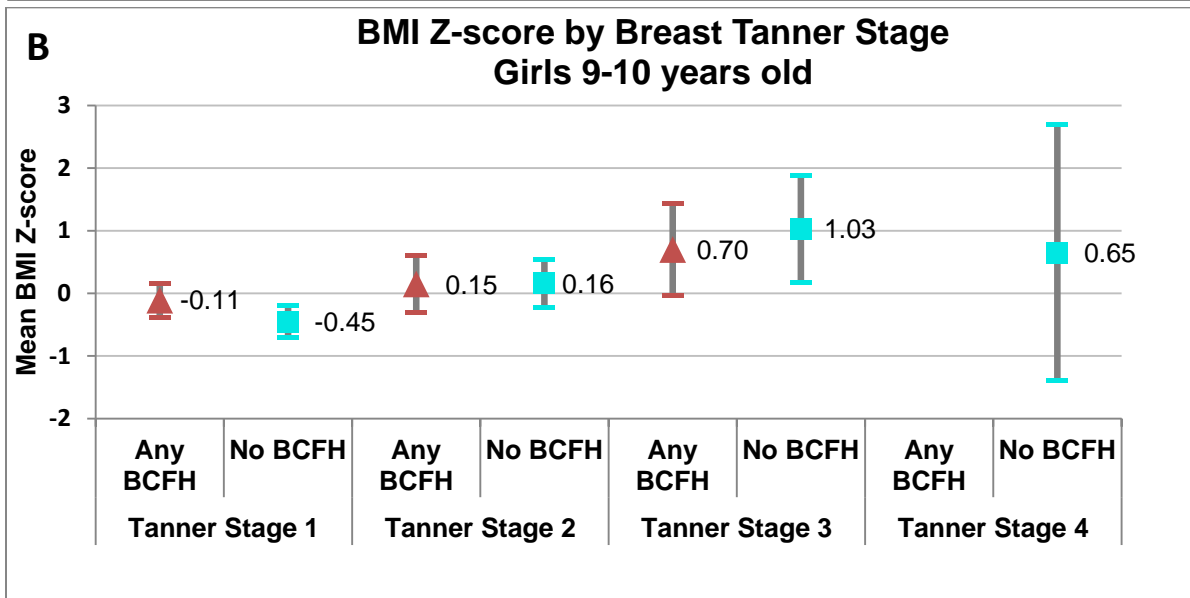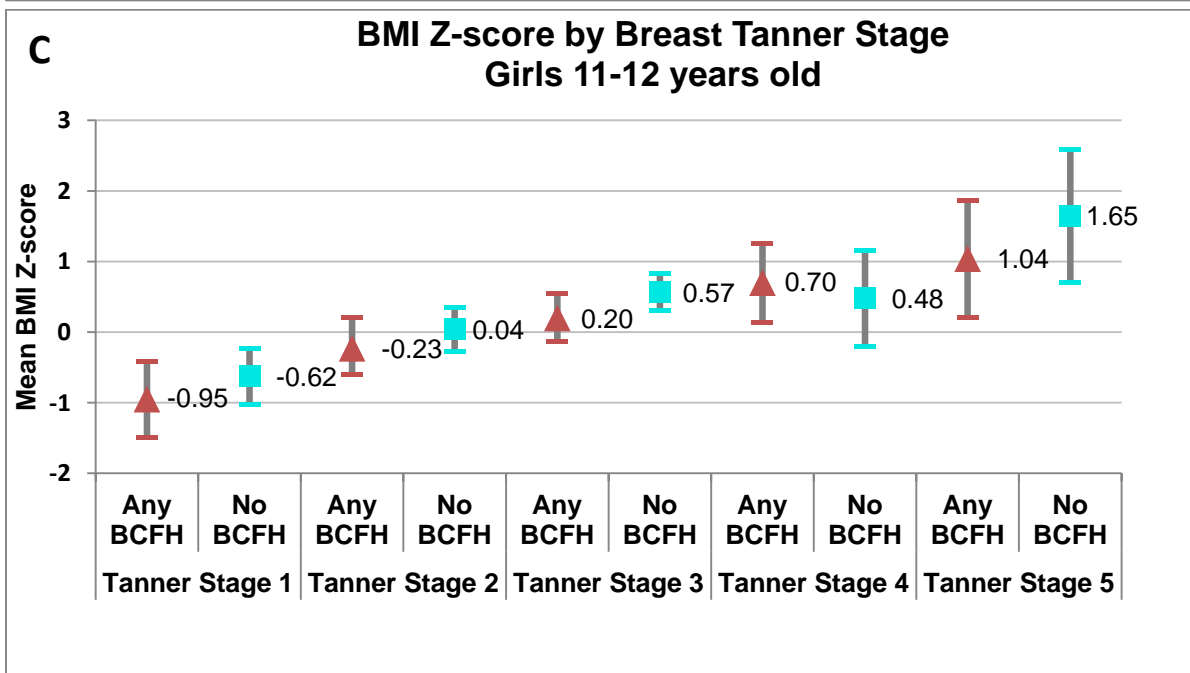

Supplement: Supplementary file 2 — Mean BMI z scores and 95% confidence intervals stratified by age group, breast Tanner stage, and breast cancer family history (BCFH). (PDF 217 kb) [file 13058_2017_849_MOESM2_ESM.pdf]
